# The Impact of Personality Traits on Mental Stress and Stigmatization in HIV+ Cases

**DOI:** 10.1192/j.eurpsy.2025.2322

**Published:** 2025-08-26

**Authors:** E. Ilgin, Ö. Yanartaş, S. Göktaş

**Affiliations:** 1 Psychiatry, Marmara University; 2 Psychology, Yeditepe University, Istanbul, Türkiye

## Abstract

**Introduction:**

HIV (Human Immunodeficiency Virus) is a virus that damages the immune system, weakening the body’s ability to defend against infections and certain types of cancer. If left untreated, HIV can progress to an advanced stage known as AIDS (Acquired Immunodeficiency Syndrome). HIV can be transmitted through blood, sexual contact, from mother to child during childbirth, or through breastfeeding. Today, with antiretroviral treatments, it is possible for individuals living with HIV to lead healthy and long lives. In addition to health issues, HIV-positive individuals face mental stress and societal stigmatization. Their personality traits play a significant role in determining the level of mental stress they experience and their ability to cope with stigma. We have developed a survey for HIV-positive individuals addressing these aspects.

**Objectives:**

This study, aimed to observe how HIV-positive individuals cope with societal stigmatization and the mental stress they experience based on their personality types, as well as the connection between these factors.

**Methods:**

The study’s survey was prepared using the open-source platform ‘Google Forms’ and will be administered in person. The tests used in this study are widely accessible and have been validated for reliability and validity in Turkey. Specifically, we utilized the Enneagram, the HIV Stigma Scale developed by Berger and colleagues, and the Hospital Anxiety and Depression Scale developed by Zigmond and Snaith. The study has no commercial purpose. The analysis was conducted on a total of 63 respondents, consisting of 45 men and 18 women.

**Results:**

In the study, data were collected from 63 individuals, 71.4% of whom were male (n=45) and 28.6% female (n=18). The average age of participants was 39.69 years (range 20-77). It was observed that individuals with primary education were the most stigmatized, while those with middle school education experienced the least stigmatization. The most common personality type among both men and women was Type 2 (the helper). According to the data, participants had an average stigma score of 94.9.

**Conclusions:**

The study did not find a significant relationship between age and stigmatization, nor between gender and stigmatization. Personality types that perceived the highest levels of stigmatization were Type 2 and Type 8, with average scores of 108. Conversely, the personality type that perceived the lowest levels of stigmatization was Type 5, with an average score of 74. These findings highlight that certain personality types may be more susceptible to experiencing or perceiving stigmatization, while others may experience it less. Further research could explore the underlying factors influencing these perceptions and their implications for support and intervention strategies.

**Disclosure of Interest:**

None Declared

